# Impact of integrating zeolite and PGPR on restoring soil health and enhancing crop yields following the leaching process

**DOI:** 10.7717/peerj.20810

**Published:** 2026-02-26

**Authors:** Mohamed A. Abd El-Aziz, Faizah Amer Altihani, Islam M. Khater, Sara A. El-Shabasy, Emad M. Hafez, Wenlong Xu, Youzhi Feng, Alaa El-Dein Omara, Tamer H. Khalifa

**Affiliations:** 1 Soil Improvement and Conservation Research Department, Soils, Water, and Environment Research Institute (SWERI), Agriculture Research Center (ARC), Giza, Egypt; 2 Department of Biology, College of Science, King Khalid University, Abha, Saudi Arabia; 3 Soil and Water Conservation Department, Desert Research Center, Cairo, Egypt; 4 Soil Chemistry and Physics Research Department, Soils, Water, and Environment Research Institute (SWERI), Agriculture Research Center (ARC), Giza, Egypt; 5 Department of Agronomy, Faculty of Agriculture, Kafrelsheikh University, Kafr El-Sheikh, Egypt; 6 Jiangsu Academy of Agricultural Sciences, Institute of Agricultural Resources and Environment, Nanjing, China; 7 Jiangsu Co-innovation Center of Efficient Processing and Utilization of Forest Resources, College of Chemical Engineering, Nanjing Forestry University, Nanjing, China; 8 Soil Microbiology Research Department, Soils, Water, and Environment Research Institute (SWERI), Agriculture Research Center (ARC), Giza, Egypt

**Keywords:** Saline soils, Zeolite, PGPR inoculation, Soil restoration, Leaching, Microbial activity, Rice, Wheat, Soil health, Sustainable approach

## Abstract

Soil leaching is a traditional approach for reclaiming saline soil; however, it often leads to nutrient loss that further weakens soil health. Therefore, this study examines the use of zeolite in combination with plant growth-promoting rhizobacteria (PGPR) as a sustainable approach to restore soil health and enhance rice and wheat yields following soil leaching. The study farm affected by salinity and sodicity is located near Kafr Basat, Dakahlia Governorate, Egypt, during the period from 2023 to 2024. A 10-week sequential leaching process (L1–L5) reduced electrical conductivity (EC) by approximately 45% and exchangeable sodium percentage (ESP) by 42% across the soil profile. However, leaching resulted in declines in soil nutrient contents and microbial activity. Four treatments: control, zeolite (Z), PGPR, and combined zeolite + PGPR (Z+PGPR), were applied using a randomized block design with three replicates. The Z+PGPR treatment significantly improved soil chemical and biological properties, including increases in cation exchange capacity (CEC) (up to 37.21 cmol kg^−1^), nitrogen, phosphorus, and potassium availability, as well as microbial biomass carbon (MBC), which increased from 4.04 to 4.37 mg/g soil. CO_2_ respiration increased by 62.3%, indicating enhanced microbial activity. Additionally, EC and ESP were further reduced, and infiltration rate improved to 1.09 cm h^−1^ under the Z+PGPR treatment. Correspondingly, rice and wheat yields increased to 11.78 t/ha and 7.44 t/ha, respectively. These results suggest that the combined application of zeolite with PGPR can enhance soil health and crop productivity following soil leaching. Nevertheless, long-term studies, multiple sites and economic assessments are needed to confirm the broader applicability and sustainability of this approach.

## Introduction

Soil salinity is a significant environmental constraint on global crop production, particularly in arid regions (Mukhopadhyay et al., 2021). Worldwide, salinity affects approximately 20% of cultivated land and 33% of irrigated land (Devkota et al., 2022). In Egypt, saline soils pose a serious threat to agricultural productivity, especially in the Nile Delta and newly reclaimed areas (Hagage et al., 2024; AbdelRahman, 2023; Aboelsoud et al., 2022). This is driven by both natural factors *e.g.*, low rainfall, high evaporation and anthropogenic activities *e.g.*, inefficient irrigation, excessive fertilizer use (El-Ramady, El-Marsafawy & Lewis, 2013). High salt concentrations in soil impair root development, limit nutrient availability and restrict water uptake, significantly reducing crop yields (Irakoze et al., 2021). Moreover, climate change-induced droughts exacerbate these issues (Tarolli et al., 2024). Salinity also deteriorates soil structure by reducing infiltration, aeration, and moisture retention (Sahab et al., 2021; Ivushkin et al., 2019).

Traditional reclamation methods, such as like gypsum application and leaching, are effective for treating saline-sodic soils (Khalifa et al., 2024; Gonçalo et al., 2020). Complementary practices, including reduced tillage, residue management, balanced fertilization, and the use of salt-tolerant crops, can further improve soil recovery (Mishra et al., 2023; El-Sharkawy et al., 2022). However, excessive tillage can degrade soil structure and deplete organic carbon (Blanco-Canqui & Ruis, 2018), while leaching often causes the loss of key nutrients (N, P, K), diminishing soil fertility (Stavi, Thevs & Priori, 2021). These challenges underscore the need for integrated, sustainable strategies that not only mitigate salinity but also promote long-term soil health.

Zeolites, microporous aluminosilicate minerals, offer a promising solution for saline soil reclamation due to their high cation exchange capacity (CEC), structural stability, and selective ion-exchange properties (Król, 2020). Their negatively charged framework, formed by the substitution of Si^4+^ by Al^3+^ in SiO_4_ and AlO_4_ tetrahedral, enables exchange of cations such as Na^+^, K^+^, Ca^2^^+^, and Mg^2^^+^ (Lang et al., 2024; Doni et al., 2020). This structure enhances nutrient retention, water-holding capacity, and fertilizer-use efficiency, critical benefits in arid environments (Pabis-Mazgaj et al., 2021; Mondal et al., 2021). Clinoptilolite, the most abundant natural zeolite, is low-cost and widely used in agricultural and wastewater applications (Senila et al., 2022). Zeolite amendments have been shown to improve hydraulic conductivity, displace sodium, and support salt-sensitive crops under saline conditions (Núñez Gómez et al., 2024; Aiad et al., 2021; Sun et al., 2020; Torres, 2016).

In saline environments, plants often experience poor germination, stunted growth, and reduced photosynthetic activity (Agbodjato & Babalola, 2024; Fasusi, Cruz & Babalola, 2021). However, plants rely on interactions with associated microbial communities, particularly plant growth-promoting rhizobacteria (PGPR) to enhance stress tolerance, nutrient acquisition, and ion homeostasis (Khalifa, Elbagory & Omara, 2021; Gamalero et al., 2020). Salt-tolerant PGPR strains achieve these benefits through the production of phytohormones (*e.g.*, auxins, cytokinins), nutrient solubilization (*e.g.*,  *via* siderophores production), and synthesis of stress-alleviating metabolites (AbuQamar et al., 2024; Cappellari et al., 2023; Tedeschi, Schillaci & Balestrini, 2023).

While controlled studies have shown that zeolites and PGPR independently mitigate salinity and enhance plant growth (Cappellari et al., 2023; Ntanos et al., 2021; Prisa, 2019), research under field conditions remains limited. These controlled experiments often fail to capture the complexity of soil–plant–microbe interactions and environmental variability of saline agricultural systems (Khalifa, Elbagory & Omara, 2021; Mondal et al., 2021). Field trials have demonstrated that zeolite amendments improve soil physicochemical properties and crop performance (Aiad et al., 2021; Sun et al., 2020). Similarly, PGPR inoculation enhances microbial activity, nutrient availability, and crop resilience to salinity (AbuQamar et al., 2024; Gamalero et al., 2020).

However, studies examining their combined application under real field conditions are scarce, and the potential synergistic benefits for soil health and crop productivity remain largely unexplored. Addressing this gap, the present study conducts a two-phase field experiment on a saline-affected farm in Egypt’s Nile Delta, providing a real-world validation of findings from controlled and pot-scale studies. The first phase evaluates the effectiveness of sequential leaching (L1–L5) in reducing soil salinity. The second examines the individual and combined applications of zeolite and PGPR for restoring soil health, enhancing microbial activity, and improving rice and wheat productivity. This integrated field approach goes beyond laboratory conditions, providing practical insights for sustainable reclamation of salt affected soils in arid regions.

## Materials & Methods

### Study site

**Figure 1:**
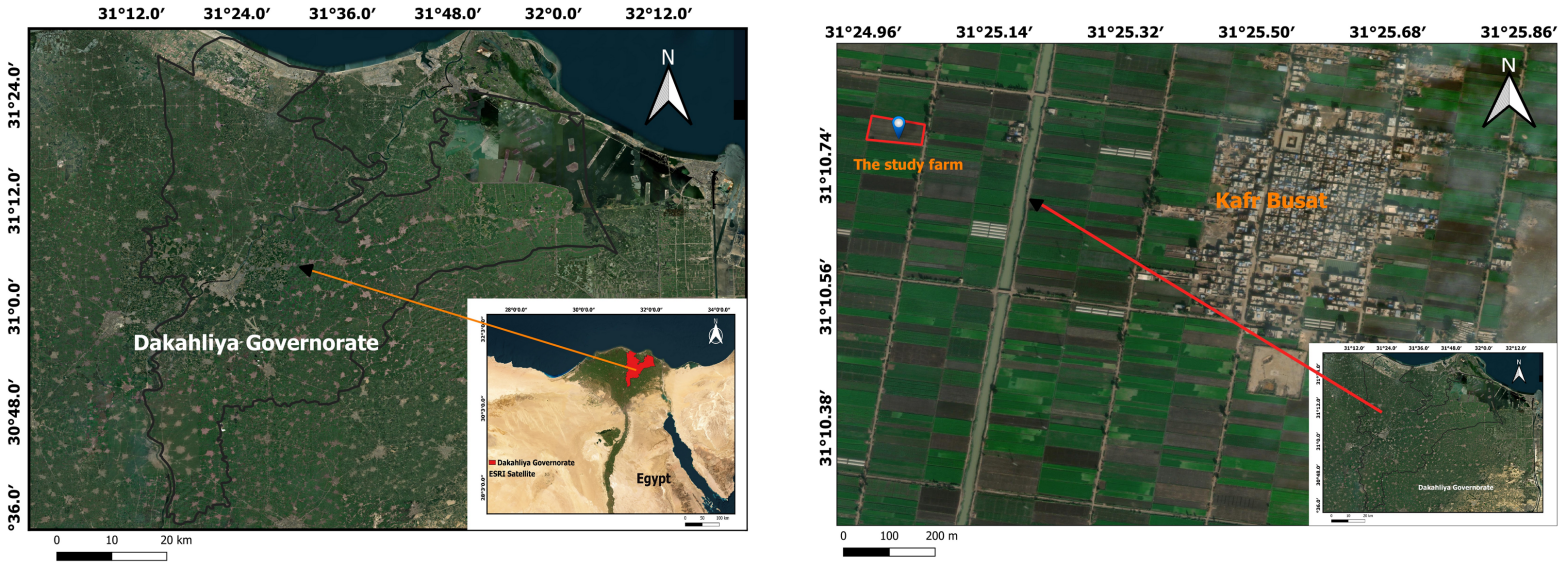
Map of the study area. The location map was created using QGIS Software (Version 3.10.1), with base map data sourced from ESRI Satellite Imagery. Map Source Credit: https://services.arcgisonline.com/ArcGIS/rest/services/World_Imagery/MapServer.

The study was conducted in Kafr Busat, located in the Dakahliya Governorate of Egypt, during the period from 2023 to 2024. The precise coordinates of the study site are 31°10′45.52″N latitude and 31°25′6.80″E longitude (Fig. 1). This region experiences a semi-arid climate, characterized by mild winters and hot summers. The average minimum temperature is 11.0 °C, while the average maximum temperature reaches 26.4 °C. Annual precipitation is extremely low, averaging just 6.5 mm, with the majority of rainfall occurring during the winter months. Evaporation rates range from 34.3 mm to 81.7 mm per year, exacerbating water scarcity in the area. The baseline soil properties are presented in Table 1.

**Table 1:** Soil properties before treated.

Soil property	Unit	Soil layer (cm)		
		0–30	30–60	60–90
Texture and Composition				
Sand	g kg^−1^	136.8	162.2	205.1
Silt	g kg^−1^	310.8	294.5	253.3
Clay	g kg^−1^	552.4	543.3	541.6
Soil Texture		Clayey	Clayey	Clayey
Chemical Properties	
pH (1:2.5 soil:water)		8.37	8.42	8.53
Electrical Conductivity (EC)	dS m^−1^	11.75	11.94	12.31
Exchangeable Sodium Percentage (ESP)	%	29.29	31.39	31.74
Cation Exchange Capacity (CEC)	cmol kg^−1^	36.94	37.43	37.62
Exchangeable Calcium (Ca^2+^)	cmol kg^−1^	8.39	7.87	7.79
Exchangeable Magnesium (Mg^2+^)	cmol kg^−1^	8.67	9.14	9.22
Exchangeable Potassium (K^+^)	cmol kg^−1^	0.73	0.75	0.76
Exchangeable Sodium (Na^+^)	cmol kg^−1^	10.82	11.75	11.94
Physical Properties	
Bulk Density	g cm^−3^	1.47	1.49	1.5
Penetration Resistance	MPa	7.95	8.82	8.99
Infiltration Rate	cm h^−1^	0.55	–	–
Aggregation Index	%	0.216	0.317	0.156
Available Soil Nutrients				
Available Nitrogen	mg kg^−1^	18.36	19.02	19.68
Available Phosphorus	mg kg^−1^	9.45	9.37	9.13
Available Potassium	mg kg^−1^	174.13	175.44	178.06
Soil Microbiological Activity	
Microbial Biomass Carbon	mg g^−1^	3.82	–	–
CO_2_ Evolution	mg CO_2_ /100 g soil	68.34	–	–

### Experimental design

The study was conducted in two phases:

1. Leaching phase: Reclaim saline-sodic soil using gypsum and repeated soil leaching monitored through sequential soil sampling over time.
2. Soil restoration phase: Evaluate the effect of soil amendments on restored soil health and enhance rice and wheat yields following soil leaching.

### Leaching phase

A 0.24-ha field (20 m × 120 m) was uniformly treated and evaluated using a within-subject (repeated measures) experimental design. Five sequential leaching events (L1–L5) were conducted at two-week intervals starting January 7, 2023. This design allowed assessment of temporal changes within the same experimental unit and was appropriate for analysis using linear mixed-effects models.

Field preparation and treatment:

- The field was sub-soiled to a depth of 90 cm using a single-share subsoiler at 5 m intervals, resulting in 4 subsoil lines across the field.
- Gypsum requirements applied at a rate of 1.27 t/field (5.28 t/ha), based on the Richards equation to reduce ESP to 15. The amendment was incorporated through deep plowing to 60 cm in two perpendicular passes.
- To control lateral water movement, two peripheral ditches (1 m deep) were constructed along the field boundaries, and internal isolation ditches were formed by sub-soiling to a depth of 90 cm.
- Leaching was performed by applying an irrigation water head of seven cm above the soil surface, delivered through intermittent irrigation cycles. The leaching water from the irrigation canal had good quality, with a pH of 7.35, EC of 0.53 dS m^−1^, and SAR of 0.48, indicating low salinity and sodicity risks for irrigation.

Following each leaching event, soil samples were collected from three depth intervals: 0–30 cm, 30–60 cm, and 60–90 cm. Samples were analyzed as a follows: infiltration rate (IR), bulk density (BD), soil aggregation index (AI) and penetration resistance (PR) and chemical analysis, electrical conductivity (EC), Exchangeable sodium percentage (ESP), cation exchange capacity (CEC), NPK availability and microbial activity.

After completion of the 10 weeks, the field was allowed to dry naturally. Peripheral ditches were closed, and the field was prepared for the next experimental phase

### Soil restoration phase

A randomized complete block design (RCBD) was used to evaluate the effects of four soil restoration treatments, with three replicates (for a total of 12 plots). The four treatments applied were:

• Control (no amendment)
• Zeolite application
• PGPR (Plant Growth-Promoting *Rhizobacteria*) inoculation
• Zeolite + PGPR (combined)

The experimental field measured 0.24 hectares (20 m × 120 m) and was divided into three blocks, each approximately six m × 40 m. Within each block, four plots (six m × nine m, or 54 m^2^) were randomly assigned to one of four treatments. Internal ditches and 1 m buffer zones were maintained between plots to minimize interference. Randomization within each block ensured unbiased allocation of treatments and helped control for spatial variability in soil properties.

### Treatment application

#### Zeolite application

A soft natural clinoptilolite zeolite from Turkey (pH: 7.10, EC: 2.24 dS/m, CEC: 158 cmol/kg, CaO: 9%, K_2_O: 4.5%, P_2_O_5_: 0.65%) was evenly incorporated into the top 20 cm of soil through plowing. The zeolite was applied in powdered form, and the application rate (640 kg/ha) was determined based on findings from previous studies and adjusted according to Aiad et al. (2021), where similar application rate under comparable saline conditions were shown to effectively improve soil physicochemical properties and enhance crop performance. These studies utilized similar soil types, and environmental conditions, providing a strong basis for applying the same rate in this study.

#### PGPR inoculation

A blend of *Bacillus subtilis* (OQ347968) and *Pseudomonas koreensis* (MG209738) in a 1:1 ratio was cultured separately in nutrient and King’s B broth media, respectively. The inoculum was prepared as a peat-based mixture, with 240 ml of each bacterial culture (10^8^ CFU/ml) added to 480 g of sterilized carrier material, resulting in a final application rate of 2.4 × 10^13^ CFU/ha. The mixture was applied to the seeds using a sticking agent and spread on a plastic sheet, kept away from direct sunlight, for a short period before sowing.

### Crop rotation

Summer season: Rice (*Oryza sativa* L., cv. Giza 178): Sown on May 6, 2023, at a rate of 120 kg/ha and harvested on September 6, 2023.

Winter season: Wheat (*Triticum aestivum* L. cv. Sakha 95): Sown on November 20, 2023, at a rate of 140 kg/ha and harvested on April 5, 2024.

#### Crops fertilization

Rice:

∘ Calcium superphosphate (15.5% P_2_O_5_) was applied at 240 kg/ha before plowing.
∘ Zinc sulfate was applied at 24.0 kg/ha before plowing.
∘ Ammonium sulfate (20.6% N) was applied in two split doses: 480 kg/ha at sowing and again one month after transplanting.
∘ Potassium sulfate (39.8% K) was applied in two split doses: 120 kg/ha at sowing and 45 days after transplanting.

Wheat:

∘ Calcium superphosphate (15.5% P_2_O_5_) was applied at 240 kg/ha before plowing.
∘ Urea (46% N) was applied in two split doses: 180 kg/ha at 30 and 60 days after sowing.
∘ Potassium sulfate (39.8% K) was applied as a single dose before sowing.

Both crops were grown to evaluate the agronomic effectiveness of the restoration treatments under real cultivation conditions.

### Measurements

#### Yield measurements

After harvesting, 12 samples (one m × one m) were collected from each treatment plot to measure grain and straw yields, which were adjusted to 18% humidity. The harvest index (HI) was calculated as the ratio of grain yield to total biomass (grain + straw).

#### Soil sampling and characterization

Soil samples were collected from three randomly selected locations within the field using a 1-meter auger (five cm diameter) at three depth intervals: 0–30 cm, 30–60 cm, and 60–90 cm during the leaching phase and at 0–30 cm after each crop in the restoration phase. Following collection, the samples were air-dried, sieved to remove large particles, homogenized and analyzed for both physical and chemical properties, as described by Carter & Gregorich (2007), Pansu & Gautheyrou (2006) and Gee & Or (2002).

Physical analysis included soil texture was performed using the pipette method and classified accordingly, infiltration rate was measured using a double-ring infiltrometer, bulk density was determined *via* the core sampler method and soil aggregation index were assessed by the wet-sieving method. Penetration resistance was measured using an electronic penetrometer (model P1.52, Eijkelkamp Agrisearch Equipment, The Netherlands), as outlined by Dexter, Czyz & Gaţe (2007).

For chemical analysis, soil pH was measured in a 1:2.5 soil-to-water suspension using a standard pH meter, while electrical conductivity (EC) was determined from saturated paste extracts using an EC meter. Exchangeable sodium percentage (ESP) was calculated, cation exchange capacity (CEC) was assessed using the ammonium saturation method, and exchangeable cations were extracted and quantified. Nutrient availability was assessed by the following methods: available nitrogen *via* the semi-micro Kjeldahl method, available phosphorus colorimetric method, and available potassium *via* flame photometry.

Microbial activity indicators included microbial biomass carbon, measured using the fumigation-extraction method Wu, Joergensen & Pommerening (1990) and calculated according to Hu, Cao & Zhiping (2007), and CO_2_ evolution, measured *via* soil incubation as described by Coleman, Anderson & Cole (1977). All analyses were conducted at the Soil Analysis Unit of SWERI under ISO-certified procedures, ensuring data accuracy and reliability.

### Statistical analysis

Data were analyzed using R (version 4.4.2). A Linear Mixed Model (LMM) was fitted using the lmer() function from the lme4 package. Treatment was included as a fixed effect, while block was treated as a random effect, with random intercepts to account for spatial variability. Analysis of variance (ANOVA) was performed, followed by Tukey’s Honest Significant Difference (HSD) test for *post-hoc* comparisons. Statistical significance was considered at *p* ≤ 0.05. Data visualization were conducted using Python 3, employing the matplotlib and seaborn libraries to generate multi-line plots and bar plots with standard division bars charts with standard deviation and significance letters

## Results

### Leaching stage

**Figure 2:**
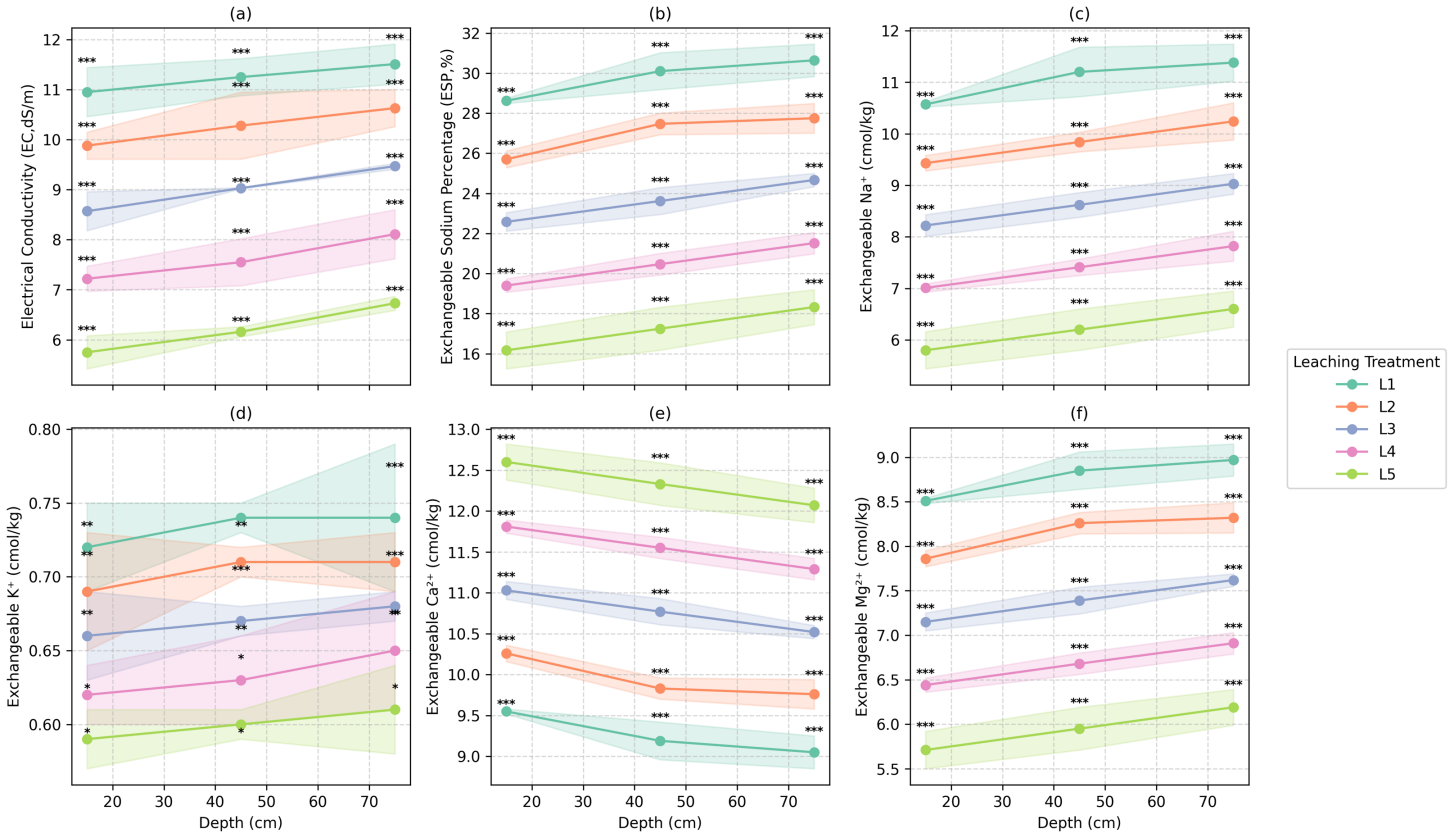
Electrical conductivity (EC), exchangeable sodium percentage (ESP), and exchangeable cations under different leaching procedures and depth. Shaded bands represent ± standard deviation for each leaching procedure across soil depths. Significant differences between treatments using Tukey’s Honestly Significant Difference test at *P* < 0.05 is indicated by marks above the shaded areas.

**Figure 3:**
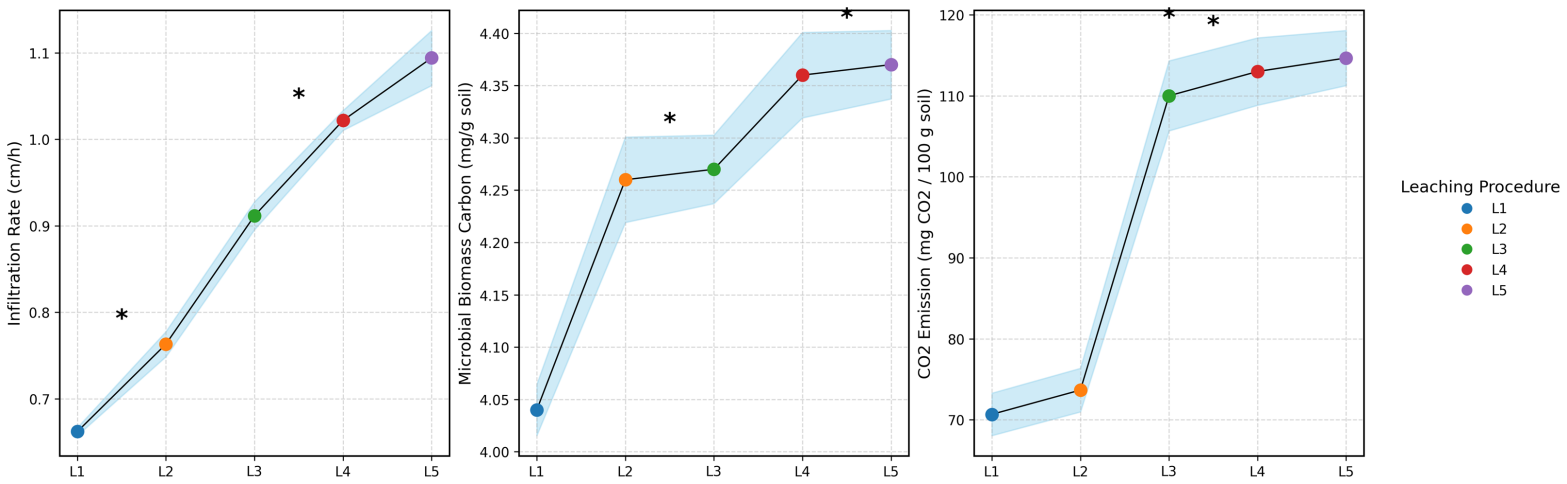
Infiltration rate (IR), microbial biomass carbon (MBC) and CO_2_ respiration (CO_2_) under different leaching procedures and depth. Shaded bands represent ± standard deviation for each leaching procedure across soil depths. Significant differences between treatments using Tukey’s Honestly Significant Difference test at *P* < 0.05 is indicated by marks above the shaded areas.

Table 2 and Figs. 2–3 present the effects of sequential leaching treatments (L1–L5) applied over a 10-week period beginning in January 2023 on key soil physicochemical properties across three depths: 0–30 cm, 30–60 cm, and 60–90 cm. Overall, improvements in soil properties were most pronounced with increasing leaching intensity, whereas depth-related changes were relatively minor.

After 10 weeks (L5), repeated leaching substantially reduced EC by approximately 45% and ESP by 42% across all soil depths (Fig. 2). The highest reductions were observed in the 0–30 cm depth, where EC declined by 47.53% and ESP dropped by 43.48%. Similar trends were observed in the 30–60 cm and the 60–90 cm layers. These results highlight the effectiveness of repeated leaching in reducing both salinity and sodicity.

**Table 2:** Some soil properties across leaching procedures and soil depths.

Leaching procedure	Depth (cm)	AI	BD	PR	CEC	Nutrient availability (mg kg^−1^)		
		(%)	(g cm^−3^)	(MPa)	cmol kg^−1^	N	P	K
L1 (2 weeks)	0–30	0.238 ± 0.04^***^	1.448 ± 0.001	6.97 ± 0.1	36.94 ± 0.18	16.89 ± 1.00	8.85 ± 0.75	173.61 ± 1.90
	30–60	0.199 ± 0.03^**^	1.473 ± 0.02	8.01 ± 0.9	37.21 ± 0.45	16.51 ± 0.73^*^	8.7 ± 0.62	173.48 ± 2.60
	60–90	0.185 ± 0.03^**^	1.491 ± 0.02^*^	8.72 ± 0.8^***^	37.14 ± 0.19	19.44 ± 0.61^***^	9.88 ± 0.38^***^	174.51 ± 2.03
L2 (4 weeks)	0–30	0.316 ± 0.01^***^	1.436 ± 0.01	8.93 ± 0.4	36.71 ± 0.09	16.75 ± 0.74	8.8 ± 0.27	173.56 ± 1.43
	30–60	0.288 ± 0.02^***^	1.452 ± 0.01	6.47 ± 0.4	35.81 ± 0.09	16.46 ± 0.55	8.68 ± 0.58	173.47 ± 1.91
	60–90	0.261 ± 0.02^***^	1.488 ± 0.01^*^	8.61 ± 0.7^***^	36.9 ± 0.31	19.69 ± 0.83^***^	9.99 ± 0.69^***^	174.6 ± 2.29
L3 (6 weeks)	0-30	0.429 ± 0.01^***^	1.419 ± 0.01	5.30 ± 1.1	36.41 ± 0.2	15.32 ± 0.48	8.22 ± 0.15	173.07 ± 2.17
	30–60	0.401 ± 0.02^***^	1.444 ± 0.02	6.81 ± 0.6	36.51 ± 0.23	14.17 ± 0.77	7.76 ± 0.19	172.66 ± 1.77
	60–90	0.374 ± 0.01^***^	1.470 ± 0.01^*^	7.87 ± 0.3^***^	36.61 ± 0.36	21.78 ± 0.88^***^	10.83 ± 0.32^***^	175.33 ± 2.83
L4 (8 weeks)	0-30	0.513 ± 0.02^***^	1.403 ± 0.01	5.11 ± 0.32	36.12 ± 0.37	14.55 ± 0.65	7.91 ± 0.45	172.8 ± 1.62
	30–60	0.485 ± 0.01^***^	1.438 ± 0.01	6.55 ± 0.52	36.22 ± 0.23	14.1 ± 0.72	7.73 ± 0.28	172.64 ± 1.77
	60–90	0.457 ± 0.01^***^	1.453 ± 0.01^*^	7.19 ± 0.51^***^	36.32 ± 0.47	21.96 ± 0.52^***^	10.91 ± 0.22^***^	175.39 ± 3.12
L5 (10 weeks)	0-30	0.568 ± 0.03^***^	1.398 ± 0.02	4.92 ± 0.87	35.83 ± 0.32	14.41 ± 0.88	7.85 ± 0.38	172.75 ± 1.34
	30–60	0.540 ± 0.03^***^	1.433 ± 0.03	6.34 ± 1.02	35.93 ± 0.24	11.57 ± 0.63	6.71 ± 0.42	171.75 ± 2.42
	60–90	0.511 ± 0.02^***^	1.448 ± 0.02^*^	6.96 ± 0.83^***^	36.02 ± 0.44	19.55 ± 0.73^***^	9.93 ± 0.53^***^	174.55 ± 3.17

**Notes.**

AI, Aggregation index; BD, Bulk Density; PR, Penetration Resistance; CEC, Cation Exchange Capacity; N, nitrogen; P, phosphorus; K, potassium. Different superscript lowercase letters in each row indicate significant differences between treatments using Tukey’s Honestly Significant Difference test at *P* < 0.05. ±Standard deviation for each leaching procedure across soil depths.

* P values: ***p < 0.001, **p < 0.01, *p < 0.05.

Improvements in other soil parameters were also observed (Table 2). Across all soil depths, the aggregation index (AI) increased by 162.16%, reflecting enhanced soil structure, Bulk density (BD) slightly decreased by 3.04%, while penetration resistance (PR) dropped by 23.49%, indicating reduced compaction and better root penetration.

Cation exchange capacity (CEC) remained stable, suggesting that leaching did not negatively affect the soil’s nutrient-holding capacity. However, significant changes were observed in exchangeable cations: sodium (Na^+^) decreased, calcium (Ca^+2^) increased, and magnesium (Mg^+2^) and potassium (K^+^) showed moderate declines (Table 2). These changes confirm successful cation exchange, with Na^+^ being replaced by more beneficial divalent cations

The impact of leaching on nutrient availability was variable. Available nitrogen (N) and phosphorus (P) exhibited non-linear responses, with slight increases in deeper layers during intermediate treatments (L3 and L4), while remained relatively stable in the surface layer. Potassium (K) was largely unaffected, likely due to its low mobility and strong binding to soil particles.

Positive trends were also observed in physical and biological parameters (Fig. 3). In the 0–30 cm layer, the infiltration rate (IR) improved significantly, indicating enhanced water movement and reduced surface sealing. Microbial activity, measured by microbial biomass carbon (MBC) and CO_2_ respiration, increased with leaching intensity. MBC slight increased from (L1) to (L5), with peak values found at deeper layers during L3 and L4. Similarly, CO_2_ respiration rates increased, reflecting improved microbial conditions under reduced salinity stress.

These findings from Phase I demonstrate that soil leaching effective in reducing salinity, sodicity and partially restoring soil conditions. However, they also reveal associated reductions in nutrients and microbial activity, highlighting the need for further restorative measures. To address this, Phase II of the study was conducted to evaluate whether soil amendments, specifically zeolite and PGPR, could complement the leaching process by restoring soil fertility, enhancing biological activity, and improving crop productivity.

### Soil restoration stage

Table 3 provides a detailed analysis of soil properties under different treatments (control, PGPR, zeolite (Z), and zeolite + PGPR) during the summer of 2023 and winter 2023/2024.

**Table 3:** Seasonal comparison of some soil properties under amendment treatments.

Seasons	Summer 2023				Winter 2024			
Variable	C^*^	PGPR^*^	Z^*^	Z+PGPR^*^	C^*^	PGPR^*^	Z ^*^	Z+PGPR^*^
Electrical conductivity (EC, dS m^−1^)	5.39 ± 0.03a	5.24 ± 0.15b	4.74 ± 0.01c	4.65 ± 0.01c	5.55 ± 0.03a	5.46 ± 0.02b	4.92 ± 0.03c	4.81 ± 0.05d
Exchangeable sodium percentage (ESP)	16.39 ± 0.19a	15.89 ± 0.10b	13.59 ± 0.22c	13.29 ± 0.07d	15.52 ± 0.16a	14.60 ± 0.57b	12.74 ± 0.22c	12.35 ± 0.13c
Infiltration rate (cm h^−1^)	1.09 ± 0.01c	1.10 ± 0.04b	1.22 ± 0.01a	1.23 ± 0.02a	1.12 ± 0.01c	1.15 ± 0.02b	1.25 ± 0.01a	1.27 ± 0.01a
Aggregation index (%)	0.563 ± 0.01c	0.576 ± 0.03b	0.667 ± 0.01a	0.675 ± 0.01a	0.586 ± 0.04c	0.61 ± 0.02b	0.69 ± 0.01a	0.70 ± 0.04a
Bulk density (g cm^−3^)	1.419 ± 0.01a	1.418 ± 0.04a	1.386 ± 0.01 b	1.381 ± 0.03b	1.415 ± 0.02a	1.422 ± 0.01a	1.377 ± 0.03b	1.370 ± 0.01b
Penetration resistance (MPa)	5.80 ± 0.18a	5.74 ± 0.18a	4.29 ± 0.47 b	4.22 ± 0.14 b	5.64 ± 0.07a	5.92 ± 0.54a	4.06 ± 0.14 b	3.80 ± 0.41b
Exchangeable calcium (Ca^2+^, cmol kg^−1^)	11.55 ± 0.05d	12.67 ± 0.03c	13.23 ± 0.06b	13.67 ± 0.24a	11.76 ± 0.04d	12.98 ± 0.14c	13.44 ± 0.06b	13.9 ± 0.25a
Exchangeable sodium (Na^+^, cmol kg^−1^)	5.78 ± 0.03a	5.69 ± 0.02a	5.33 ± 0.05b	5.30 ± 0.03 c	5.62 ± 0.03a	5.35 ± 0.19b	5.15 ± 0.052c	5.12 ± 0.03c
Cation exchangeable capacity (CEC, cmol kg^−1^)	35.27 ± 0.20d	35.81 ± 0.11c	39.25 ± 0.27b	39.86 ± 0.11a	36.20 ± 0.17d	36.65 ± 0.13c	40.44 ± 0.31b	41.44 ± 0.42a

**Notes.**

* C, without amendments; PGPR, soil + PGPR; Z, soil + zeolite; Z+PGPR, soil + PGPR + zeolite. Different superscript lowercase letters in each row indicate significant differences between treatments using Tukey’s Honestly Significant Difference test at *P* < 0.05. ±Standard deviation for each treatments.

The treatments with zeolite (Z) and the combined zeolite + PGPR (Z+PGPR) showed significantly reduced electrical conductivity (EC) and exchangeable sodium percentage (ESP) compared to the control and PGPR-only treatments (Table 3).

Infiltration rate (IR) and aggregation index (AI) were significantly higher in the Z and Z+PGPR treatments, indicating improved soil structure. Bulk density (BD) and penetration resistance (PR) were significantly lower in Z and Z+PGPR treatments, suggesting reduced soil compaction. Additionally, CEC and Ca^2+^ levels were higher, while Na^+^was lower in Z and Z+PGPR treatments (Table 3).

**Figure 4:**
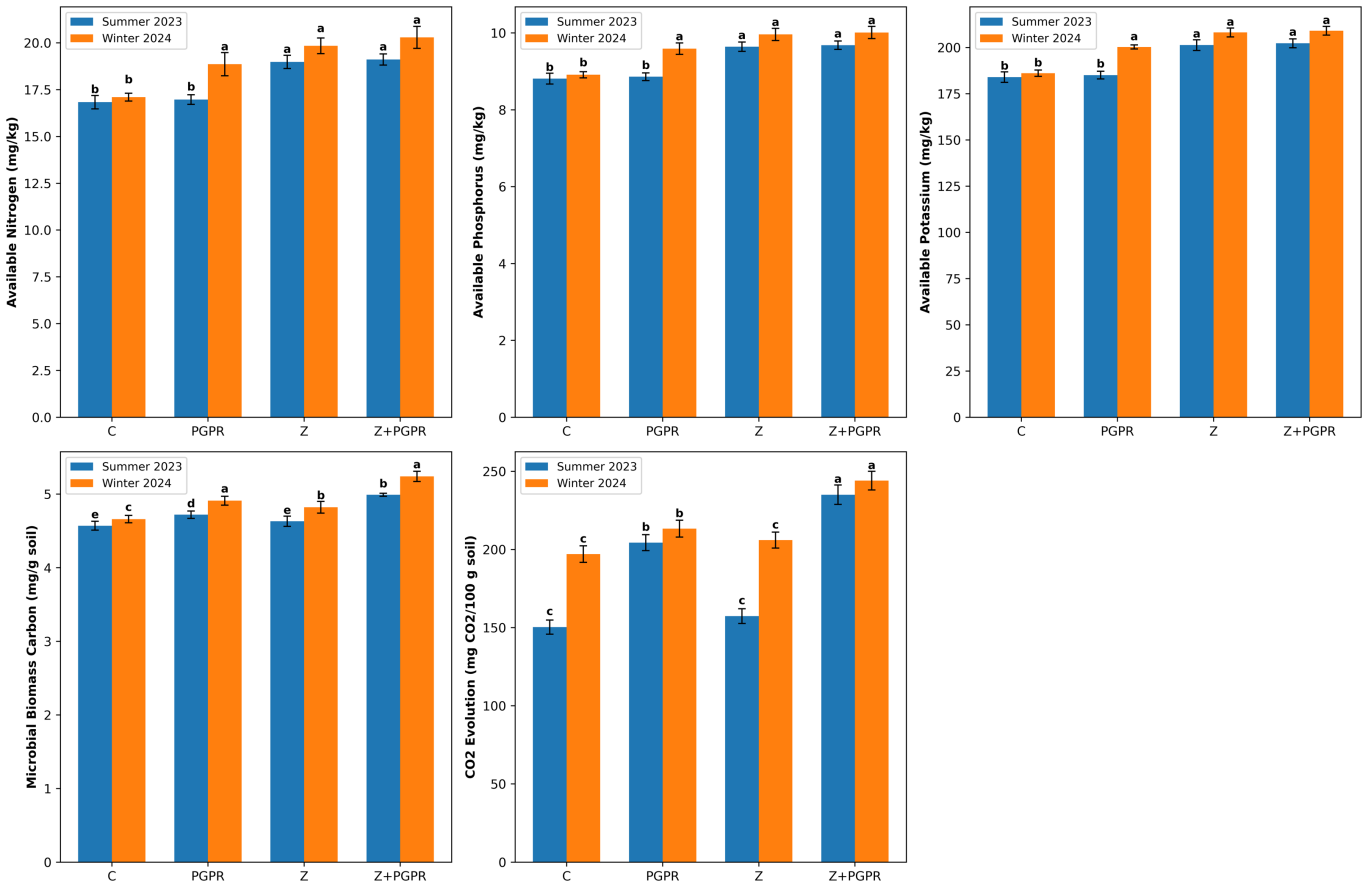
Seasonal comparison of available soil nutrients and soil microbiological activity under amendment treatments. C, without amendments; PGPR, soil + PGPR; Z, soil + zeolite; Z + PGPR, soil + PGPR + zeolite. Different lowercase letters above bar indicate significant differences between treatments using Tukey’s Honestly Significant Difference (HSD) test) at *P* < 0.05. ±Standard deviation for each treatments.

Nutrient availability was also enhanced. Nitrogen (N), phosphorus (P), and potassium (K) was significantly higher in the Z and Z+PGPR treatments compared to the control and PGPR-only treatments, indicating improved soil fertility (Fig. 4).

Microbial activity, measured by microbial biomass carbon (MBC) and CO_2_ respiration, was significantly higher in the PGPR and Z+PGPR treatments relative to the control and Z-only treatments, demonstrating increased microbial activity (Fig. 4).

Overall, the results from both phases support the central hypothesis: leaching alone effectively reduces salinity (EC) and sodicity (ESP), it must be complemented by soil amendments to fully restore soil fertility and biological health. Phase I established a reclaimed baseline, reducing salinity and sodicity related constraints and improving physical properties such as infiltration and aggregation, but with a trade-off in microbial activity and nutrient levels, particularly nitrogen and phosphorus. Phase II demonstrated that applying zeolite and PGPR, especially in combination, effectively replenished nutrients, improved soil physical structure, and revitalized microbial activity, creating a more favorable environment for crop growth and soil sustainability.

### Crop yield and harvest index

**Figure 5:**
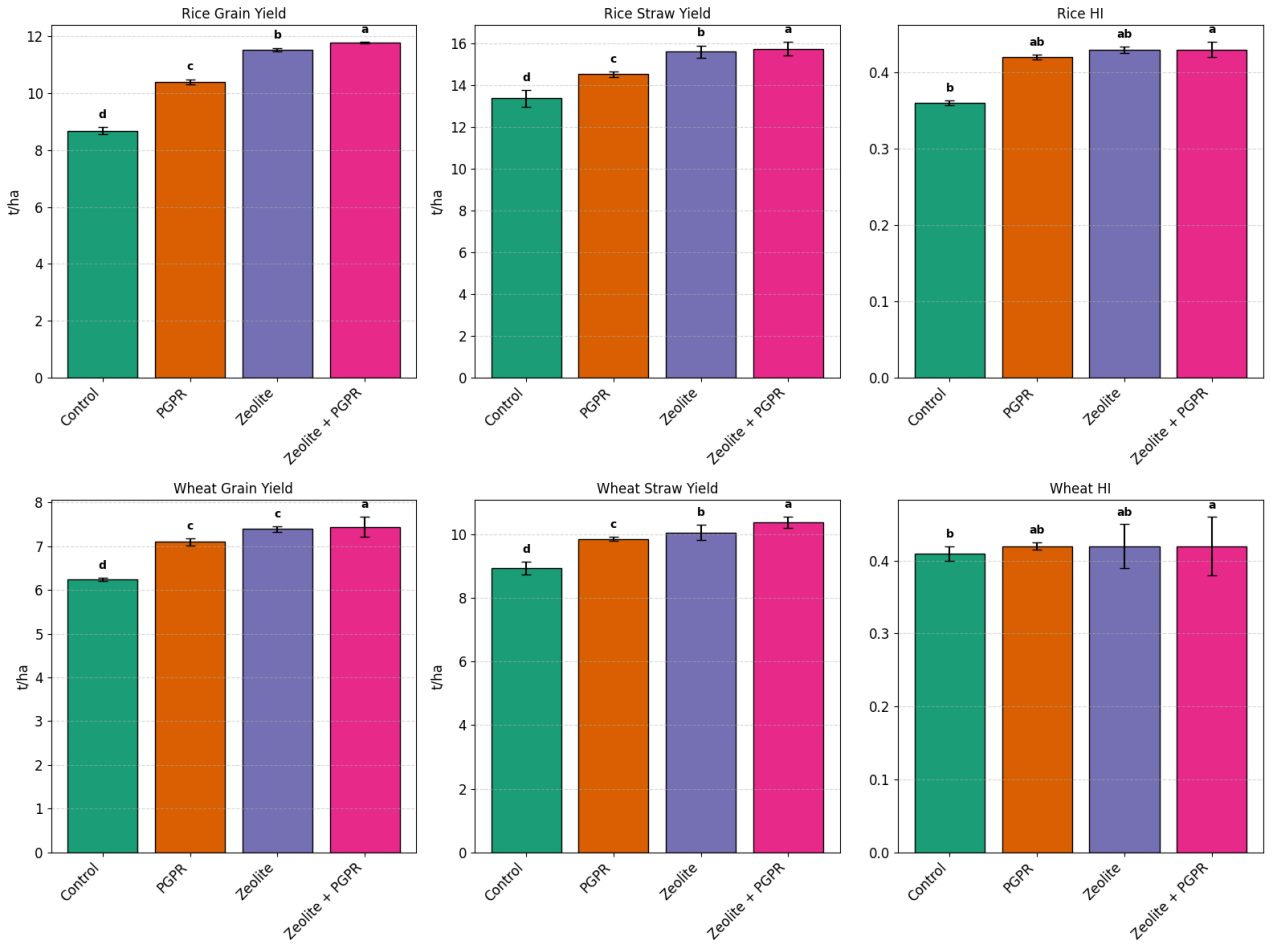
Crop Yield and Harvest index under amendment treatments. C, without amendments; PGPR, soil + PGPR; Z, soil + zeolite; Z+PGPR, soil + PGPR + zeolite. Different lowercase letters above bar indicate significant differences between treatments using Tukey’s Honestly Significant Difference (HSD) test) at *P* < 0.05. ±Standard deviation for each treatments.

The data in Fig. 5 present a detailed analysis of crop yield and harvest index (HI) for rice (summer 2023) and wheat (winter 2023/2024) under different treatments (Control, PGPR, Z, Z+PGPR), illustrating the impact of the amendments on crop productivity. The lowest grain yields were observed in the control treatment (C), with 8.69 t/ha for rice and 6.24 t/ha for wheat. The highest grain yields were recorded in the combined zeolite + PGPR treatment, with 11.78 t/ha for rice and 7.44 t/ha for wheat. Treatments with PGPR and zeolite alone produced intermediate grain yields for both crops (Fig. 5).

A similar trend was observed for straw yield, with the lowest values in the control treatment and the highest in the Z+PGPR treatment (Fig. 5). The lowest Harvest Index (HI) values were recorded for both rice and wheat in the control treatment (Fig. 5). The highest HI values occurred in the Z+PGPR treatment, indicating improved allocation of resources towards grain production. However, the HI values for both crops in Z+PGPR treatments were not significantly different from those of the Z or PGPR treatments alone, suggesting that the combined application provides only marginal additional benefits for these crops (Fig. 5).

Overall, these results support the hypothesis that the combined use of zeolite and PGPR can enhance both soil health and crop performance following soil leaching. The synergistic improvements in soil structure, nutrient availability, and microbial activity translated directly into increased crops productivity.

## Discussion

### Effectiveness of leaching procedures

Under the studied conditions, high salinity, sodicity, and soil compaction posed significant limitations to plant growth and overall soil health. These constraints impeded water infiltration, restricted root development, and limited nutrient availability, underscoring the need for effective soil reclamation strategies.

Leaching, a key method for managing saline-sodic soils, effectively reduced electrical conductivity (EC) and exchangeable sodium percentage (ESP), particularly in intensive treatments (L4 and L5). These treatments facilitated the downward movement of soluble salts and exchangeable Na^+^, improved soil structure by increasing Ca^2+^ and enhanced soil aggregation. These findings are consistent with (Khalifa et al., 2024; Sahab et al., 2021; Ivushkin et al., 2019; Qadir et al., 2007), who emphasized soil leaching and gypsum addition are effective in improving soil quality by removing excess salts and sodium ions. Additionally, sub-soiling was employed to reduce compaction and improve water movement by enhancing soil structure (Stavi, Thevs & Priori, 2021; Cuevas et al., 2019).

Leaching also increased infiltration rates and soil aggregation indices, further improving soil physical properties such as reduced bulk density and penetration resistance. Although a direct causal relationship between ion exchange and infiltration improvement was not tested, the observed reductions in Na^+^ and increases in Ca^2+^ and aggregation indices suggest that leaching can enhance soil permeability and structure. As noted by Zhang et al. (2024), these improvements were most pronounced in the upper soil layers, where sodium removal alleviated compaction, creating conditions more favorable for root development and water retention. However, the loss of soluble nutrients, particularly nitrogen and phosphorus, may affect long-term soil fertility (Stavi, Thevs & Priori, 2021; Krofft et al., 2020). Potassium levels remained stable, likely due to its stronger adsorption to soil particles (Goulding et al., 2021).

Microbial activity showed moderate improvement with increased leaching intensity, as indicated by microbial biomass carbon (MBC) and CO_2_ respiration. This suggests that alleviating salinity stress supports better microbial conditions, aligning with findings by Yan & Marschner (2013). However, the limited gains in microbial indicators suggest that leaching alone may not fully restore biological soil health, and additional interventions may be necessary.

The findings from Phase I demonstrate that leaching effectively reduces soil EC and ESP, thereby partially restoring soil conditions. Physical properties, such as infiltration rates and aggregation indices improved due to the flushing of excess salts and sodium from the soil profile. However, these benefits were accompanied by losses of essential nutrients, particularly N and P, and only modest improvements in microbial activity, highlighting a critical limitation: leaching alone is insufficient to fully restore soil fertility and biological function.

To address these shortcomings, Phase II of the study evaluated whether soil amendments, specifically zeolite and PGPR, could complement the leaching process. These amendments aimed to restore nutrient levels, enhance microbial activity, and further improve soil structure and crop productivity.

### Impact of zeolite and PGPR on soil and crop productivity

Following leaching, the application of zeolite (Z) and plant growth-promoting rhizobacteria (PGPR), especially in combination (Z+PGPR), further improved soil health. These findings support previous reports that Z and PGPR can reduce salinity and sodicity and enhance soil quality (Aiad et al., 2021; Doni et al., 2020; Prisa, 2019). However, the mechanisms were inferred rather than directly tested and should be interpreted cautiously.

Zeolite’s high cation exchange capacity (CEC) may contribute to the adsorption of sodium, replacing it with calcium, which may explain the observed reductions in EC and ESP (Mondal et al., 2021; Aiad et al., 2021; Doni et al., 2020; Prisa, 2019; Khalifa et al., 2019). This hypothesis is supported by the observed increases in CEC, Ca^2+^ in Z and Z+PGPR treatments (Torres, 2016). Enhanced water retention, nutrient availability, and improved soil aggregation contributed to more favorable conditions for plant growth (Núñez Gómez et al., 2024). However, since this mechanistic pathway was inferred rather than directly tested in this study and should be interpreted with caution.

Soil physical properties improved under Z and Z+PGPR treatments, with higher infiltration rate (IR), increased aggregation index (AI), reduced bulk density (BD) and lower penetration resistance (PR), indicating improved porosity and reduced compaction (Gholizadeh-Sarabi & Sepaskhah, 2013; Mondal et al., 2021).

Nutrient availability improved, with increased levels of nitrogen (N), phosphorus (P), and potassium (K). Zeolite likely enhanced nutrient retention, while PGPR supported nutrient uptake *via* N fixation and P solubilization (AbuQamar et al., 2024; Tedeschi, Schillaci & Balestrini, 2023).

The combination of amendments Z and PGPR stimulated microbial activity, as evidenced by higher MBC and CO_2_ respiration, indicating improved biological soil health (Naseri et al., 2022). Initial findings suggest that Z may enhance PGPR survival in saline soils, while PGPR may improve nutrient uptake in zeolite-treated soils (Ntanos et al., 2021; Prisa, 2019). However, these relationships remain correlational, and further studies are needed to understand the underlying mechanisms.

Seasonal variation also influenced the results. Soil properties in winter 2023/2024 were generally improved relative to summer, likely due to cooler temperatures and higher soil moisture, which favor microbial growth and nutrient transport (Uzel, Stanton & Scott, 2023; Elbasiouny et al., 2022). Given that this study was conducted over a single season, the results should be considered short-term, and long-term multi-year studies are needed to validate these findings.

The Z+PGPR treatment produced the highest grain and straw yields for both rice and wheat, along with an improved HI, indicating more efficient resource allocation to grain production. These results are consistent with prior studies showing that PGPR improves nutrient uptake and Z enhances nutrient and water retention (Khalifa et al., 2019; Chen et al., 2017; Torres, 2016). Grain and straw yields under Z+PGPR were significantly higher than in other treatments, while the HI also improved, although the added benefit over individual treatments was modest, suggesting potential crop-specific responses (Win, Oo & Yokoyama, 2022; Aiad et al., 2021; Sun et al., 2020).

It should be noted that these findings are based on a short-term, single-site study. While this design strengthens internal validity by controlling site-specific factors, it limits the generalization of results to other locations or longer-term conditions.

## Conclusions

This study tested two hypotheses: (1) sequential leaching reduces soil salinity and sodicity and improves soil physical characteristics; (2) combining zeolite + PGPR after leaching further improves soil fertility, microbial activity, and crop yields.

The results that leaching substantially reduced EC and ESP at all depths and improved infiltration, aggregation index, bulk density, and other physical indices, providing strong support for the first hypothesis. The second hypothesis is partially supported: soils treated with zeolite + PGPR showed higher nutrient availability, greater microbial biomass, and increased crop yields than control and single-treatment plots. Applying 640 kg/ha of zeolite with PGPR may therefore be a promising strategy for managing saline-sodic soils in arid and semi-arid regions.

These findings are based on a short-term, single-site study with only one control treatment. Future studies should include additional controls, such as a gypsum-only plots, to improve comparison. Furthermore, the economic feasibility and scalability of this approach remain untested. Long-term, multi-site trials and cost-benefit analyses are needed to confirm the sustainability and broader applicability of this strategy.

## Supplemental Information

10.7717/peerj.20810/supp-1 Supplemental Information 1 Data analysis 3 Linear Mixed Model (LMM) statistical analysis of soil physical and chemical properties across leaching procedures and soil depths during the leaching stage.

10.7717/peerj.20810/supp-2 Supplemental Information 2 Data analysis 2 Linear Mixed Model (LMM) statistical analysis of microbial activity indicators and soil infiltration rate across leaching procedures and soil depths during the leaching stage.

10.7717/peerj.20810/supp-3 Supplemental Information 3 Data analysis 4 Linear Mixed Model (LMM) statistical analysis of soil properties and crop yields under different zeolite and PGPR treatments during the restoring stage.

10.7717/peerj.20810/supp-4 Supplemental Information 4 Leaching stage raw data Raw experimental data collected during the leaching stage, including soil physical, chemical, and biological measurements.

10.7717/peerj.20810/supp-5 Supplemental Information 5 Raw experimental data collected during the restoring stage, including soil properties and crop yield parameters under zeolite and PGPR treatments.

## Additional Information and Declarations

### Data Availability

The following information was supplied regarding data availability: The raw data is available in the Supplemental Files.
