# Supplementary material for: Impact of integrating zeolite and PGPR on restoring soil health and enhancing crop yields following the leaching process
Source: PeerJ. 2026 Feb 26;14:e20810. doi: 10.7717/peerj.20810 (PMC12950185; doi:10.7717/peerj.20810)
Supplement: Supplemental Information 1 — Linear Mixed Model (LMM) statistical analysis of soil physical and chemical properties across leaching procedures and soil depths during the leaching stage. [file peerj-14-20810-s001.pdf]

	EC			ESP			AI			BD		
Predictors	*Estimates*	*std. Error*	*p*	*Estimates*	*std. Error*	*p*	*Estimates*	*std. Error*	*p*	*Estimates*	*std. Error*	*p*
(Intercept)	11.24	0.27	**<0.001**	29.79	0.65	**<0.001**	0.21	0.02	**<0.001**	1.47	0.01	**<0.001**
Leaching Procedure [L2]	-0.98	0.19	**<0.001**	-2.81	0.35	**<0.001**	0.07	0.01	**<0.001**	0.00	0.01	0.820
Leaching Procedure [L3]	-2.21	0.19	**<0.001**	-6.16	0.35	**<0.001**	0.19	0.01	**<0.001**	-0.03	0.01	**0.022**
Leaching Procedure [L4]	-3.61	0.19	**<0.001**	-9.32	0.35	**<0.001**	0.28	0.01	**<0.001**	-0.04	0.01	**0.001**
Leaching Procedure [L5]	-5.02	0.19	**<0.001**	-12.53	0.35	**<0.001**	0.33	0.01	**<0.001**	-0.04	0.01	**<0.001**
**Random Effects**												
σ^2	0.16			0.56			0.00			0.00		
τ_{00}	0.16 $_{Soil.Depth..cm.}$			1.07 $_{Soil.Depth..cm.}$			0.00 $_{Soil.Depth..cm.}$			0.00 $_{Soil.Depth..cm.}$		
ICC	0.49			0.66			0.66			0.37		
N	3 $_{Soil.Depth..cm.}$			3 $_{Soil.Depth..cm.}$			3 $_{Soil.Depth..cm.}$			3 $_{Soil.Depth..cm.}$		
Observations	45			45			45			45		
Marginal R^2 / Conditional R^2	0.912 / 0.955			0.926 / 0.975			0.932 / 0.977			0.309 / 0.566		

	PR			Ca			Mg			K		
Predictors	*Estimates*	*std. Error*	*p*	*Estimates*	*std. Error*	*p*	*Estimates*	*std. Error*	*p*	*Estimates*	*std. Error*	
(Intercept)	7.90	0.56	**<0.001**	9.26	0.16	**<0.001**	8.78	0.15	**<0.001**	0.73	0.01	[illegible]
Leaching Procedure [L2]	0.10	0.48	0.830	0.69	0.09	**<0.001**	-0.63	0.08	**<0.001**	-0.03	0.00	[illegible]
Leaching Procedure [L3]	-1.24	0.48	**0.014**	1.51	0.09	**<0.001**	-1.39	0.08	**<0.001**	-0.07	0.00	[illegible]
Leaching Procedure [L4]	-1.62	0.48	**0.002**	2.28	0.09	**<0.001**	-2.10	0.08	**<0.001**	-0.10	0.00	[illegible]
Leaching Procedure [L5]	-1.83	0.48	**0.001**	3.07	0.09	**<0.001**	-2.83	0.08	**<0.001**	-0.13	0.00	[illegible]
**Random Effects**												
σ^2	1.04			0.03			0.03			0.00		
τ_{00}	0.61 $_{Soil.Depth..cm.}$			0.06 $_{Soil.Depth..cm.}$			0.05 $_{Soil.Depth..cm.}$			0.00 $_{Soil.Depth..cm}$		
ICC	0.37			0.66			0.66			0.66		
N	3 $_{Soil.Depth..cm.}$			3 $_{Soil.Depth..cm.}$			3 $_{Soil.Depth..cm.}$			3 $_{Soil.Depth..cm.}$		
Observations	45			45			45			45		
Marginal R^2 / Conditional R^2	0.291 / 0.551			0.926 / 0.975			0.926 / 0.975			0.926 / 0.975		
