# Supplementary material for: Impact of integrating zeolite and PGPR on restoring soil health and enhancing crop yields following the leaching process
Source: PeerJ. 2026 Feb 26;14:e20810. doi: 10.7717/peerj.20810 (PMC12950185; doi:10.7717/peerj.20810)
Supplement: Supplemental Information 2 — Linear Mixed Model (LMM) statistical analysis of microbial activity indicators and soil infiltration rate across leaching procedures and soil depths during the leaching stage. [file peerj-14-20810-s002.pdf]

	MBC			CO2			IR		
Predictors	*Estimates*	*std. Error*	*p*	*Estimates*	*std. Error*	*p*	*Estimates*	*std. Error*	*p*
(Intercept)	4.04	0.02	**<0.001**	70.67	2.48	**<0.001**	0.66	0.02	**<0.001**
Leaching [L2]	0.22	0.01	**<0.001**	3.00	0.79	**0.005**	0.10	0.01	**<0.001**
Leaching [L3]	0.23	0.01	**<0.001**	39.33	0.79	**<0.001**	0.25	0.01	**<0.001**
Leaching [L4]	0.32	0.01	**<0.001**	42.33	0.79	**<0.001**	0.36	0.01	**<0.001**
Leaching [L5]	0.33	0.01	**<0.001**	44.00	0.79	**<0.001**	0.43	0.01	**<0.001**
**Random Effects**									
σ^2	0.00			0.95			0.00		
τ_{00}	0.00 $_{Rep}$			17.52 $_{Rep}$			0.00 $_{Rep}$		
ICC	0.96			0.95			0.94		
N	3 $_{Rep}$			3 $_{Rep}$			3 $_{Rep}$		
Observations	15			15			15		
Marginal R^2 / Conditional R^2	0.893 / 0.996			0.958 / 0.998			0.962 / 0.998		
