# Supplementary material for: Impact of integrating zeolite and PGPR on restoring soil health and enhancing crop yields following the leaching process
Source: PeerJ. 2026 Feb 26;14:e20810. doi: 10.7717/peerj.20810 (PMC12950185; doi:10.7717/peerj.20810)
Supplement: Supplemental Information 3 — Linear Mixed Model (LMM) statistical analysis of soil properties and crop yields under different zeolite and PGPR treatments during the restoring stage. [file peerj-14-20810-s003.pdf]

	EC			ESP			IR			AI			BD			PR		
Predictors	*Estimates*	*std. Error*	*p*	*Estimates*	*std. Error*	*p*	*Estimates*	*std. Error*	*p*	*Estimates*	*std. Error*	*p*	*Estimates*	*std. Error*	*p*	*Estimates*	*std. Error*	*p*
(Intercept)	5.39	0.03	**<0.001**	16.39	0.15	**<0.001**	1.09	0.01	**<0.001**	0.56	0.00	**<0.001**	1.42	0.00	**<0.001**	5.80	0.18	**<0.001**
Seasons [Winter 2023/2024]	0.16	0.04	**0.002**	-0.87	0.13	**<0.001**	0.03	0.00	**<0.001**	0.02	0.00	**<0.001**	-0.00	0.00	0.509	-0.17	0.23	0.474
Amendments [PGPR]	-0.15	0.04	**0.002**	-0.51	0.13	**0.002**	0.02	0.00	**0.003**	0.01	0.00	**0.013**	-0.00	0.00	0.509	-0.06	0.23	0.784
Amendments [Z]	-0.65	0.04	**<0.001**	-2.81	0.13	**<0.001**	0.14	0.00	**<0.001**	0.10	0.00	**<0.001**	-0.03	0.00	**<0.001**	-1.51	0.23	**<0.001**
AmendmentsZ+PGPR	-0.74	0.04	**<0.001**	-3.10	0.13	**<0.001**	0.15	0.00	**<0.001**	0.11	0.00	**<0.001**	-0.04	0.00	**<0.001**	-1.58	0.23	**<0.001**
Seasons [Winter 2023/2024] × Amendments [PGPR]	0.06	0.06	0.317	-0.42	0.19	**0.044**	0.02	0.01	**0.024**	0.01	0.01	0.156	0.01	0.01	0.173	0.35	0.32	0.297
Seasons [Winter 2023/2024] × Amendments [Z]	0.03	0.06	0.652	0.02	0.19	0.903	0.00	0.01	1.000	-0.00	0.01	0.625	-0.01	0.01	0.354	-0.06	0.32	0.846
Seasons [Winter 2023/2024] × AmendmentsZ+PGPR	0.00	0.06	0.955	-0.07	0.19	0.716	0.00	0.01	0.622	0.00	0.01	0.625	-0.01	0.01	0.354	-0.26	0.32	0.430
**Random Effects**																		
σ^2	0.00			0.03			0.00			0.00			0.00			0.08		
τ_{00}	0.00_{Rep}			0.04_{Rep}			0.00_{Rep}			0.00_{Rep}			0.00_{Rep}			0.02_{Rep}		
ICC	0.32			0.59			0.64			0.47			0.21			0.22		
N	3_{Rep}			3_{Rep}			3_{Rep}			3_{Rep}			3_{Rep}			3_{Rep}		
Observations	24			24			24			24			24			24		
Marginal R^2 / Conditional R^2	0.969 / 0.979			0.970 / 0.988			0.981 / 0.993			0.978 / 0.988			0.909 / 0.928			0.885 / 0.911		

	Ca			Na			CEC			AN			AP			AK		
Predictors	*Estimates*	*std. Error*	*p*	*Estimates*	*std. Error*	*p*	*Estimates*	*std. Error*	*p*	*Estimates*	*std. Error*	*p*	*Estimates*	*std. Error*	*p*	*Estimates*	*std. Error*	*p*
(Intercept)	11.54	0.08	**<0.001**	5.78	0.04	**<0.001**	35.27	0.14	**<0.001**	16.83	0.57	**<0.001**	8.81	0.22	**<0.001**	183.97	4.55	**<0.001**
Seasons [Winter 2023/2024]	0.22	0.09	**0.036**	-0.16	0.05	**0.009**	0.93	0.10	**<0.001**	2.03	0.67	**0.009**	0.78	0.26	**0.009**	16.33	5.35	**0.009**
Amendments [PGPR]	1.13	0.09	**<0.001**	-0.09	0.05	0.098	0.54	0.10	**<0.001**	0.14	0.67	0.841	0.05	0.26	0.838	1.10	5.35	0.841
Amendments [Z]	1.69	0.09	**<0.001**	-0.45	0.05	**<0.001**	3.98	0.10	**<0.001**	2.15	0.67	**0.006**	0.83	0.26	**0.006**	17.31	5.35	**0.006**
AmendmentsZ+PGPR	2.13	0.09	**<0.001**	-0.48	0.05	**<0.001**	4.59	0.10	**<0.001**	2.28	0.67	**0.004**	0.88	0.26	**0.004**	18.29	5.35	**0.004**
Seasons [Winter 2023/2024] × Amendments [PGPR]	0.10	0.13	0.476	-0.18	0.07	**0.032**	-0.09	0.14	0.521	-1.90	0.95	0.064	-0.73	0.36	0.063	-15.29	7.57	0.063
Seasons [Winter 2023/2024] × Amendments [Z]	-0.01	0.13	0.941	-0.02	0.07	0.792	0.26	0.14	0.084	-1.18	0.95	0.234	-0.45	0.36	0.232	-9.46	7.57	0.232
Seasons [Winter 2023/2024] × AmendmentsZ+PGPR	0.02	0.13	0.901	-0.02	0.07	0.792	0.65	0.14	**<0.001**	-0.85	0.95	0.384	-0.46	0.36	0.228	-9.52	7.57	0.229
**Random Effects**																		
σ^2	0.01			0.00			0.02			0.67			0.10			43.01		
τ_{00}	0.01_{Rep}			0.00_{Rep}			0.04_{Rep}			0.31_{Rep}			0.04_{Rep}			19.07_{Rep}		
ICC	0.29			0.29			0.73			0.31			0.31			0.31		
N	3_{Rep}			3_{Rep}			3_{Rep}			3_{Rep}			3_{Rep}			3_{Rep}		
Observations	24			24			24			24			24			24		
Marginal R^2 / Conditional R^2	0.974 / 0.981			0.905 / 0.933			0.989 / 0.997			0.631 / 0.747			0.614 / 0.732			0.614 / 0.733		

	Grain			Straw			HI			MBC			CO2		
Predictors	*Estimates*	*std. Error*	*p*	*Estimates*	*std. Error*	*p*	*Estimates*	*std. Error*	*p*	*Estimates*	*std. Error*	*p*	*Estimates*	*std. Error*	*p*
(Intercept)	3.59	0.06	**<0.001**	9.95	0.14	**<0.001**	0.36	0.01	**<0.001**	4.57	0.03	**<0.001**	150.33	3.07	**<0.001**
Seasons [Winter 2023/2024]	0.70	0.06	**<0.001**	0.91	0.19	**<0.001**	0.04	0.01	**<0.001**	0.34	0.03	**<0.001**	63.00	0.48	**<0.001**
Amendments [PGPR]	0.71	0.06	**<0.001**	1.15	0.19	**<0.001**	0.03	0.01	**0.003**	0.13	0.03	**0.002**	54.00	0.48	**<0.001**
Amendments [Z]	1.18	0.06	**<0.001**	2.23	0.19	**<0.001**	0.03	0.01	**0.001**	0.06	0.03	0.101	7.00	0.48	**<0.001**
AmendmentsZ+PGPR	1.28	0.06	**<0.001**	2.37	0.19	**<0.001**	0.04	0.01	**<0.001**	0.42	0.03	**<0.001**	84.67	0.48	**<0.001**
Seasons [Winter 2023/2024] × Amendments [PGPR]	-0.41	0.09	**<0.001**	-0.84	0.27	**0.008**	-0.01	0.01	0.227	-0.22	0.05	**<0.001**	-61.33	0.68	**<0.001**
Seasons [Winter 2023/2024] × Amendments [Z]	-0.78	0.09	**<0.001**	-1.72	0.27	**<0.001**	-0.02	0.01	0.136	-0.31	0.05	**<0.001**	-23.33	0.68	**<0.001**
Seasons [Winter 2023/2024] × AmendmentsZ+PGPR	-0.47	0.09	**<0.001**	-1.44	0.27	**<0.001**	-0.00	0.01	0.756	-0.09	0.05	0.083	-54.00	0.68	**<0.001**
**Random Effects**															
σ^2	0.01			0.05			0.00			0.00			0.35		
τ_{00}	0.01_{Rep}			0.01_{Rep}			0.00_{Rep}			0.00_{Rep}			28.01_{Rep}		
ICC	0.51			0.12						0.48			0.99		
N	3_{Rep}			3_{Rep}			3_{Rep}			3_{Rep}			3_{Rep}		
Observations	24			24			24			24			24		
Marginal R^2 / Conditional R^2	0.945 / 0.973			0.897 / 0.909			0.834 / NA			0.931 / 0.964			0.972 / 1.000		
